# Paroxysmal Atrial Fibrillation Catheter Ablation of Pulmonary Veins: does Anatomy Influence the Outcome?

**DOI:** 10.5935/abc.20180229

**Published:** 2018-12

**Authors:** Álvaro Valentim Lima Sarabanda

**Affiliations:** Instituto de Cardiologia do Distrito Federal - Fundação Universitária de Cardiologia (FUC), Brasília, DF - Brazil

**Keywords:** Heart Failure, Arrhythmias, Cardiac, Catheter Ablation, Pulmonary Veins

Atrial fibrillation (AF) is the most common cardiac arrhythmia found in clinical practice and a frequent cause of hospital admission. AF is associated with a 5-fold increased risk of stroke and an approximately 2-fold increased risk of death, in addition to also being associated with heart failure development.^1^

Approximately 20 years ago, the percutaneous radiofrequency ablation of the pulmonary veins (PVs) was described by Haissaguerre et al.^2^ as an effective technique and curative treatment of paroxysmal AF (PAF). The initial technique of AF ablation was developed based on the observation that the electrical activity triggers (ectopic foci), responsible for causing PAF, are frequently located in the PVs. As a consequence, the initiation of PAF could be prevented through the ablation of these triggers.^2^

Subsequently, aiming to prevent potential procedure complications, such as PV stenosis, and also to improve its success rates, the PV ablation procedure was progressively modified, from the PV focal ablation technique to the segmental electrical isolation of the PV ostia, resulting in the predominant present-day technique of extended circumferential antral ablation of PVs (1 to 2 cm extended area from the PV ostia).^1,3^

Most of the available data^1,3^ indicate that the circumferential antral ablation of the PVs is more effective than the ostial ablation of the PVs. The beneficial mechanisms of PV circumferential antral ablation are not fully established but are probably related to the isolation of the triggers in the PV antrum, the modification of the ganglionated plexi, or the interruption of AF initiation and/or maintenance mechanisms located within the PV antrum.^1,3^

The most frequent PV circumferential antral ablation technique uses radiofrequency energy, delivered point-by-point through an external irrigated-tip catheter, with the help of a three-dimensional, electroanatomical mapping system as a navigation guide and also for the creation of a visual record of the ablated sites.

More recently, irrigated catheters have become available, with contact force-sensing technology, which is able to measure the contact force intensity between the tip of the catheter and the myocardium, increasing the effectiveness of the radiofrequency ablation lesion in the myocardium, and reducing procedure complication rates.^1^ Cryoablation, which uses a balloon-catheter to attain PV isolation, is an equally validated alternative technique.^1^

Currently, the shortcoming of the PV circumferential ablation is the AF recurrence during the first year after the ablation, an event typically related to the electrical reconnection of PVs to the left atrium.^1^ Therefore, several lines of research are focused on identifying techniques and procedures that can provide a permanent electrical isolation of the PVs during the initial AF ablation procedure.

In this context, in the current issue of the Arquivos Brasileiros de Cardiologia, Odozynski et al.,^4^ report the results of circumferential antral ablation of the PVs in PAF treatment, specifically comparing patients who had a common trunk of the left PVs (CTrL) versus those without CTrL.

An electroanatomical mapping system based on chest impedance was used in all procedures and patients underwent circumferential isolation of the VP antrum by delivering radiofrequency with an irrigated-tip catheter but without monitoring the contact force, aiming at obtaining entrance and exit block into the PVs.

In the present study, in agreement with the world's literature, approximately 17% of the patients had a CTrL. It should be emphasized that during the medium-term clinical follow-up, a lower recurrence rate of AF was observed in patients with CTrL when compared to patients without CTrL.^4^

The current study has the merit of providing a timely overview of the complexity found in the present-day percutaneous ablation of PVs, discussing the implications that PV anatomy can have on PAF ablation outcome. As reported in the study, four pulmonary veins reach the left atrium in most patients. The CTrL, defined as the fusion of the 2 left PVs into a common trunk, is the most common anatomical variation of the PVs, occurring in 4% to 18% of patients undergoing AF ablation.^5^

As pointed out by the authors, the possible reason for patients with CTrL to show a lower recurrence rate of PAF could be related to the fact that it is easier to handle and attain better contact between the ablation catheter and the left atrium in patients presenting with CTrL.^4^ As previously reported,^1,3^ the intensity of the contact force between the ablation catheter and the myocardium is crucial for the radiofrequency lesion formation and has been associated with longer-lasting PV isolation and better clinical outcomes.

Furthermore, as already has been discussed, circumferential antral ablation of the PVs is more effective than the ostial ablation of the PVs, probably related to the isolation of the triggers in the antrum of the PVs, modification of the ganglionated plexi, or the interruption of AF initiation and/or maintenance mechanisms located within the PV antrum.
